# 17β-estradiol regulates giant vesicle formation via estrogen receptor-alpha in human breast cancer cells

**DOI:** 10.18632/oncotarget.1824

**Published:** 2014-03-15

**Authors:** Paul K Wright, Sarah Bowen Jones, Nicholas Ardern, Rebecca Ward, Robert B Clarke, Federica Sotgia, Michael P Lisanti, Goran Landberg, Rebecca Lamb

**Affiliations:** ^1^ Department of Histopathology, Manchester Royal Infirmary, Central Manchester University Hospitals NHS Foundation Trust, Manchester, UK; ^2^ Manchester Cytology Centre, Manchester Royal Infirmary, Central Manchester University Hospitals NHS Foundation Trust, Manchester, UK; ^3^ Breakthrough Breast Cancer Research Unit, University of Manchester, Manchester, UK; ^4^ Breast Biology Group, University of Manchester, Manchester, UK; ^5^ Sahlgrenska Cancer Center, University of Gothenburg, Sweden

**Keywords:** Breast cancer, vesicle, estrogen, trafficking, exocytosis

## Abstract

A significant proportion of the genes regulated by 17-beta-estradiol (E2) via estrogen receptor alpha (ERα) have roles in vesicle trafficking in breast cancer. Intracellular vesicle trafficking and extracellular vesicles have important roles in tumourigenesis. Here we report the discovery of giant (3-42μm) intracellular and extracellular vesicles (GVs) and the role of E2 on vesicle formation in breast cancer (BC) cell lines using three independent live cell imaging techniques. Large diameter vesicles, GVs were also identified in a patient-derived xenograft BC model, and in invasive breast carcinoma tissue. ERα-positive (MCF-7 and T47D) BC cell lines demonstrated a significant increase in GV formation after stimulation with E2 which was reversed by tamoxifen. ERα-negative (MDA-MB-231 and MDA-MB-468) BC cell lines produced GVs independently of E2 and tamoxifen. These results indicate the existence of both intracellular and extracellular vesicles with considerably larger dimensions than generally recognised with BC cells and suggest that the GVs are regulated by E2 via ERα in ERα-positive BC but by E2-independent mechanisms in ER-ve BC.

## INTRODUCTION

Estrogens, in particular 17-beta-estradiol (E2), are a fundamental driving force in the development of breast cancer and act via estrogen receptor-alpha (ERα) to regulate the expression of select genes [1-5]. The proteins encoded by the estrogen-regulated transcriptome are considered to act in concert to promote breast tumourigenesis. Abrogation of E2-induced gene expression by anti-estrogens and aromatase inhibitors forms the basis of endocrine therapy for breast cancer

Vic *et al* described an ultrastructural study of breast cancer, including evidence that E2 regulates secretion and an exocytosis-like process in MCF-7 cells [6]. Of the large number of estrogen-regulated genes (~8,000) identified by DNA microarray studies, 147 transcripts have recently been implicated in vesicle trafficking including exocytosis in breast cancer cell lines [7] indicating that a significant proportion of the estrogen-regulated transcriptome regulates vesicle trafficking in breast cancer cells. Frasor *et al* identified the vesicle trafficking genes RAB31 and RAB30 as E2 and tamoxifen-regulated respectively [8]. Gene expression analysis of breast carcinoma samples from patients treated with anastrozole show differential expression of vesicle trafficking genes in non-responders compared with responders, suggesting that vesicle trafficking may be involved in anastrozole resistance [9].

Recent evidence indicates that vesicle trafficking, including exocytosis and endocytosis, has important roles in tumourigenesis (10-13). The translocation breakpoint (t11:22 (p13;q12)) of desmoplastic small round cell tumour produces a chimeric transcription factor (EWS-WT1) shown to induce BAIAP3, a protein implicated in exocytosis, providing evidence supporting a role for exocytosis in cancer [14-15]. Many other vesicle trafficking genes, have been related to cancer [10-13] and breast cancer including annexin A1, claudin 7, RAB3A, RAB5A, RAB6C, RAB8, RAB11-FIP, RAB25, RAB27A, RAB27B, RAB31 and RAB38 [16-29]. RAB27B regulates invasive tumour growth and metastasis in ER-positive breast cancer cell lines and xenograft murine models. Furthermore, RAB27B mRNA and protein expression is associated with lymph node metastasis and tumour grade in ER-positive tumours [28].

Additional support for a role of vesicles in cancer is provided by the large body of evidence that microvesicles (MV) mediate of tumourigenesis [10,30-35]. MVs are membrane-bound vesicles that are released from many types of normal cells as well as cancer cells. They are considered to exert their effects as ectoorganelles by acting as paracrine or endocrine signalling vehicles but are generally reported to be up to 1μm in diameter. [14,30-35].

Here we report a novel type of intracellular and extracellular vesicles that we term giant vesicles (GV). To capture the morphology of breast cancer cells optimally, we used three independent live cell imaging techniques. The most striking finding was the identification of novel large intracellular and extracellular vesicles that were up to 42μm in diameter in breast cancer cell lines, invasive breast carcinoma tissue samples and primary xenograft tumour samples. We show that E2 induces and tamoxifen represses GV formation in ERα-positive breast cancer cell lines (MCF-7 and T47D) and that GVs are produced by ERα-negative breast cancer cell lines (MDA-MB-231 and MDA-MB-468) in an E2-independent manner. However, giant vesicle formation became E2-dependent in MDA MB-231 cells on expression of ERα protein suggesting that E2 induces giant vesicle formation via ERα.

## RESULTS

### Validation of Giant Vesicles (GVs) in breast cancer cells

**Figure 1:**
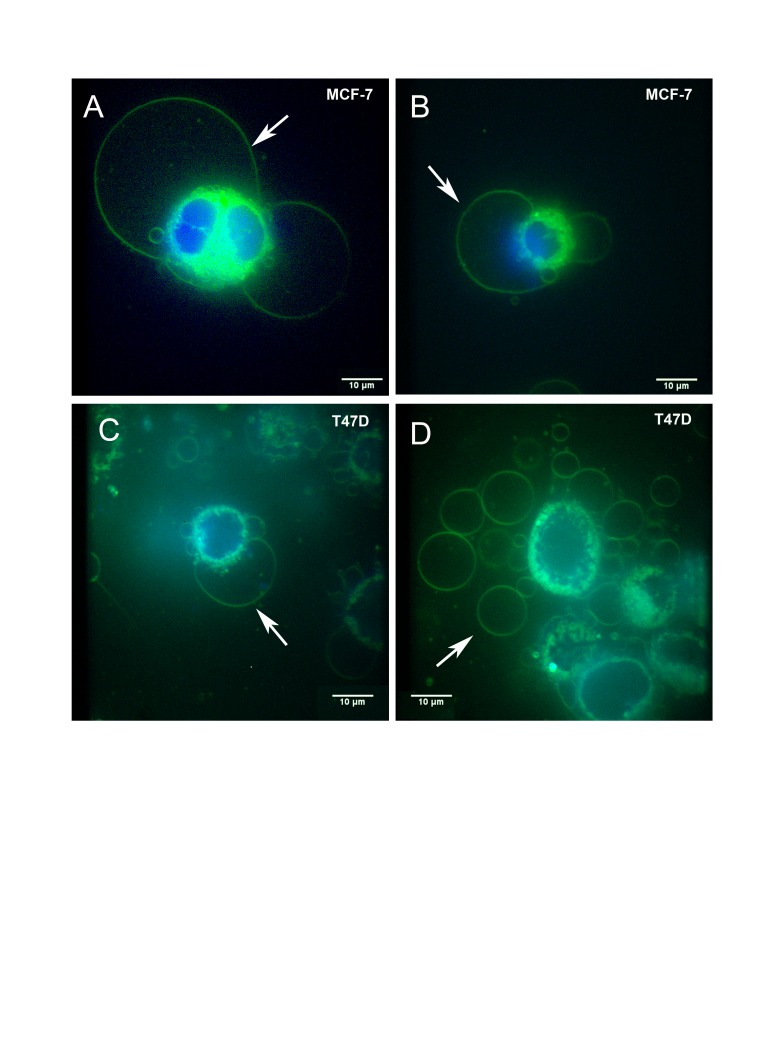
Live cell FM® 1-43FX fluorescent imaging identifies giant vesicles in breast cancer cells MCF-7 (1A and 1B) and T47D (1C and 1D) cells were cultured and labelled with FM® 1-43FX (green) to stain intracellular giant vesicles (1A-C) and extracellular giant vesicles (1D). Cells were also labelled with DAPI (blue) to stain the nucleus. Figure 1A illustrates two intracellular giant vesicles, the larger measuring 40.04μm in diameter. Each of these intracellular giant vesicles contains a smaller internal vesicle. Figure 1A also demonstrates cytoplasmic fluorescence produced by FM® 1-43FX which is present as a fluorescent green rim surrounding the nucleus. Giant vesicles showed fluorescence within their outer membrane and within the membranes of internal vesicles. No fluorescence was observed from within the giant vesicles. Figure 1D illustrates a large number of extracellular giant vesicles in the extracellular fluid in the same field as T47D cells. Imaging was performed using an Olympus IX81 with spinning disk confocal microscope. Scale bar represents 10µm.

Live cell imaging using the fluorescent non-toxic lipophilic styryl dye FM® 1-43FX, labelled the membranes of large intracellular vesicles in breast cancer cell lines (MCF7 and T47D) cultured under standard conditions (Figure 1A-D). Intracellular and extracellular vesicles were 3-42μm in diameter. Nuclei were labelled with DAPI to define the subcellular architecture including the spatial relationship of intracellular GV to the nucleus. DAPI labelling demonstrated nuclear fluorescence as expected and no fluorescence within intracellular or extracellular GV, confirming that GV were not separate or dividing cells (Figure 1A-D). GV were identified in breast cancer cells with FM® 1-43FX-labelling alone (data not shown), confirming that the presence of GV was unrelated to the effects of DAPI.

Intracellular GV were localised to the periphery of breast cancer cells (Figure 1A-C). Although some cells had a single eccentrically placed intracellular GV but many had multiple (up to six) intracellular GV that were present at the periphery of the cell (Figure 1A-C). The contents of GV were shown to be non-fluorescent other than some considerably smaller internal vesicles (Figure 1A) demonstrating that, at least some GV, are multivesicular bodies. In view of the lipophilic nature of FM® 1-43FX and FM® 4-64FX dyes, the general lack of fluorescence within GV suggests that their contents are aqueous.

FM® 1-43FX is endocytosed into the cytoplasm producing intense cytoplasmic fluorescence which allowed the spatial relationship of GV to the cell cytoplasm to be demonstrated. The cytoplasm was limited to an approximately spherical region surrounding the nucleus with an architecture that would be expected based on our current knowledge in cell biology and diagnostic cytopathology (Figure 1A-C). Most of the GV membrane was surrounded by extracellular fluid and not cytoplasm (Figure 1A-C). Where present, the GV membrane formed part of the outer limiting membrane of the cell.

**Figure 2:**
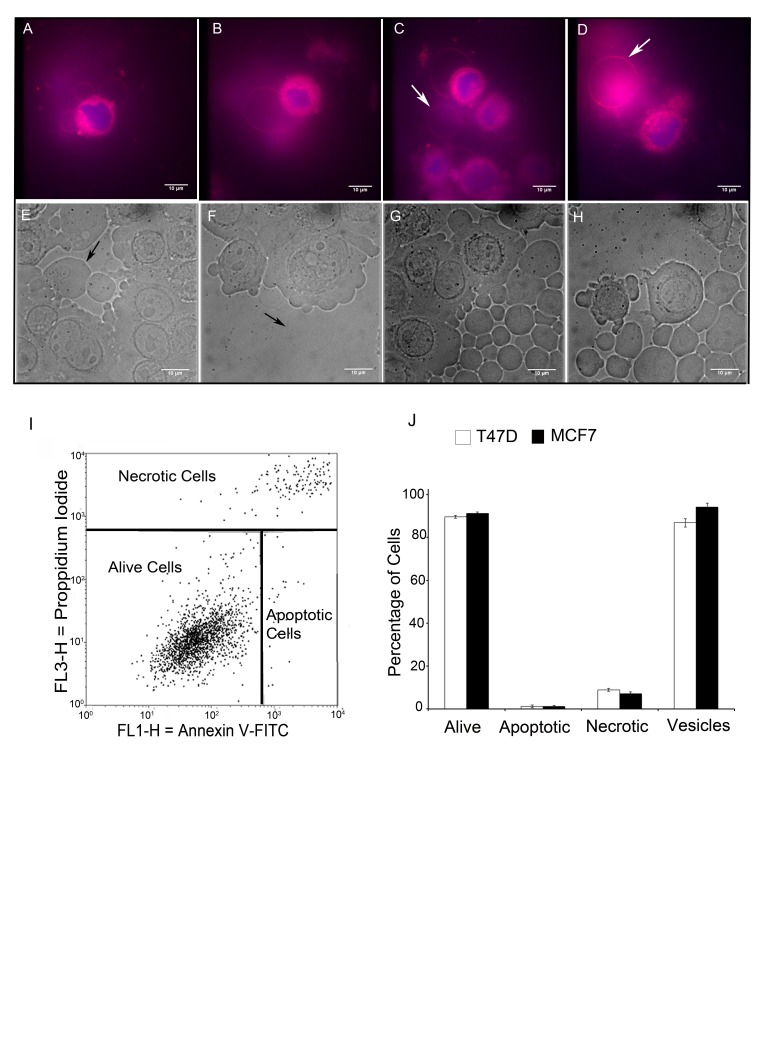
Live cell FM® 4-64FX fluorescent imaging and differential contrast microscopy imaging identify non-apoptotic giant vesicles MCF-7 cells were cultured and labelled with FM® 4-64FX (red) and DAPI (blue) and imaged using an Olympus CKX41 inverted fluorescence microscope (2A-D). FM® 4-64FX red fluorescence labelled the membranes of intracellular giant vesicles (2A-B) and extracellular giant vesicles (2C-D: arrows indicate extracellular giant vesicles). (2E-H) Differential Interference Contrast (DIC) microscopy was performed using a Zeiss Axiovert 200M inverted microscope. DIC identified intracellular giant vesicles (2E-F: arrows indicate intracellular vesicles) and extracellular giant vesicles (2G-H). Scale bar represents 10µm. (I) The percentage of apoptotic/necrotic cells in MCF-7 cells as determined by Annexin V-FITC /Propidium Iodide analysis. (J) Bar chart comparison of the percentage of alive, apoptotic and necrotic cells to the percentage of cells producing GV in MCF-7 and T47D cells with +- SE bars.

The identification of GV with FM® 1-43FX labelling was validated by using two further independent live cell imaging techniques. A second lipophilic styryl dye FM® 4-64FX was used to label membranes in the MCF-7 breast cancer cell line. This identified similar large intracellular and extracellular vesicles in MCF-7 breast cancer cells (Figure 2A-D). We also used differential interference contrast microscopy (DIC) to image live T47D and MCF-7 breast cancer cells that were mounted in PBS alone without any labelling to exclude the possibility of artefacts produced by fluorescent styryl dyes or DAPI. DIC imaging identified similar large intracellular and extracellular vesicles in both cell lines (Figure 2E-H).

The possibility that GV formation could be a phenomenon related to apoptosis or necrosis was investigated by imaging live cells with FM® 1-43FX and DAPI in conjunction with Annexin V/Propidium Iodide FACS analysis to determine rates of apoptosis and necrosis. This demonstrated that cultured T47D and MCF-7 cells had 90% viability and that GV were produced by over 85% of cells. The small numbers of necrotic and apoptotic cells, excluded the possibility that these processes gave rise to GV (Figure 2I and 2J).

**Figure 3:**
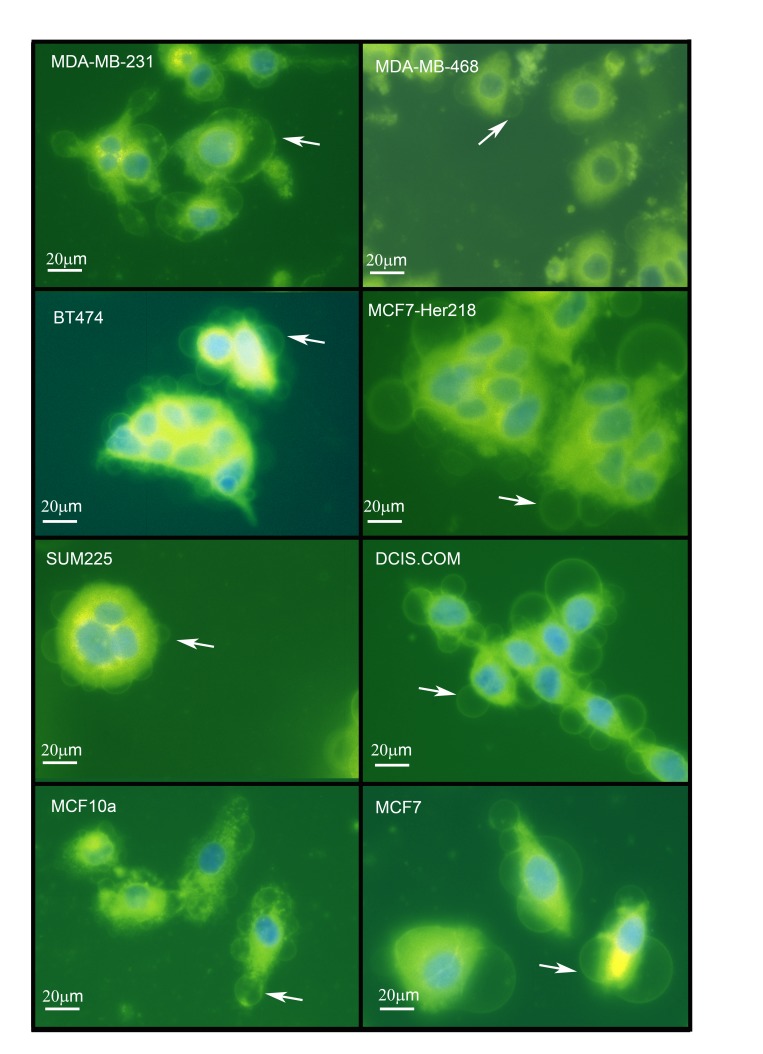
Giant vesicles are produced by a wide range of breast cancer cell lines and by MCF10A breast epithelial cells Cell lines (MDA-MB-231, MDA-MB-468 [ER-negative], BT-474, MCF7-HER218 [HER2-positive], SUM225, DCIS.com [DCIS], MCF10A [“normal” breast] and MCF-7 [ER-positive] were cultured and labelled with FM® 1-43FX (green) and DAPI (blue) before imaging using an Olympus CKX41 inverted fluorescence microscope. Scale bar represents 20µm.

**Figure 4:**
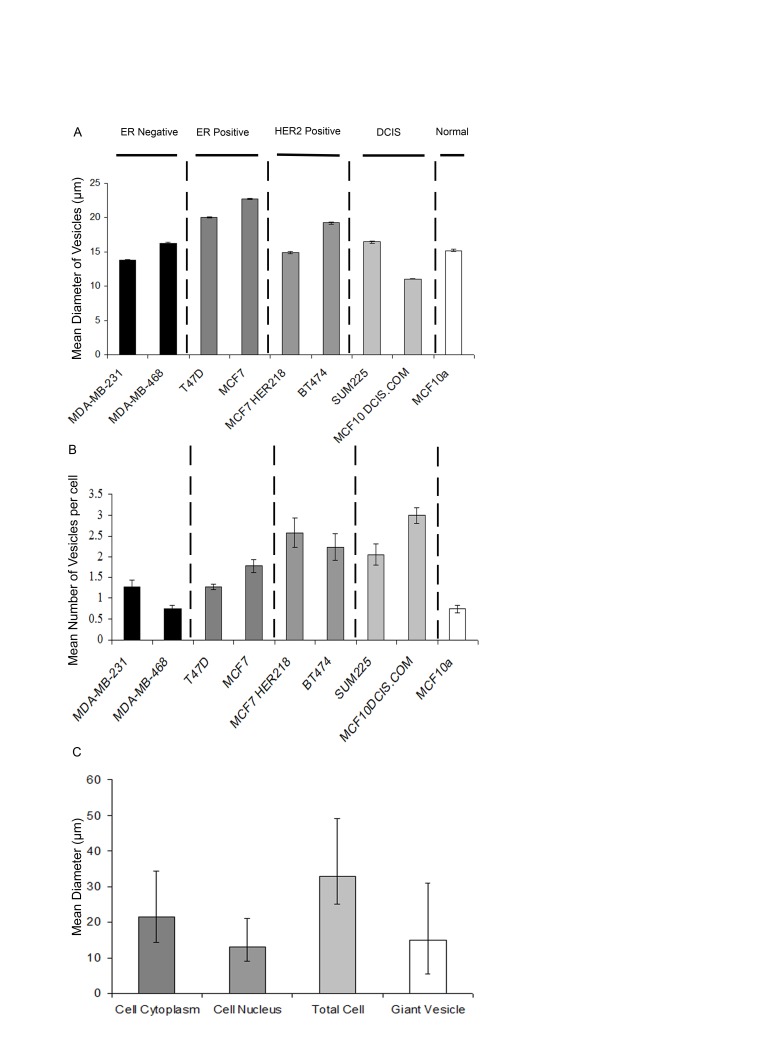
Quantification of Giant vesicle production in breast cancer cell lines and MCF10A breast epithelial cells Cell lines (MDA-MB-231, MDA-MB-468 (ER-negative), BT-474, MCF7-HER218 (HER2-positive), SUM225, DCIS.com (DCIS), MCF10A (“normal” breast) and MCF-7 and T47D (ER-positive) were cultured and labelled with FM® 1-43FX (green) and DAPI (blue) before imaging using an Olympus CKX41 inverted fluorescence microscope. Vesicle diameter (µm) was measured within thirteen fields of view (x400 magnification) and the mean calculated with +- SE bars (4A). The number of vesicles produced per cell was also counted within 13 fields of view (x400 magnification) and the mean calculated with +- SE bars (4B). Nuclear diameter, cytoplasm diameter, total cell diameter and giant vesicle diameter were measured in 20 representative MCF7 cells and the means calculated with error bars added to represent the range (maximum and minimum) in diameter observed (4C).

We performed FM® 1-43FX and DAPI labelling to image live cells in a total of eight breast cancer cell lines and in one non-neoplastic breast epithelial cell line (MCF-10A) grown under standard conditions. This identified intracellular GV with similar morphological features to that described above in all nine cell lines (Figure 3). This suggests that intracellular GV are a widespread phenomenon in cultured breast cancer cells in addition to being present in a non-neoplastic breast epithelial cell line. The diameter of intracellular GV ranged from 3 to 42μm in the nine cell lines. The mean diameters of GV in the nine cell lines including MCF10A were similar. However, there was some variation between cell lines with the largest mean GV diameter (23µm)in MCF-7 cells and the lowest mean diameter (11µm) in MCF10/DCIS.com cells (Figure 4A).

Subsequently, the number of intracellular GV per cell was counted in the nine cell lines. This demonstrated that intracellular GV are a dominant feature in these cell lines and are not limited to a minority of cells (Figure 4B). A variation between different cell lines was identified, with the lowest number in MCF-10A cells (mean 0.7 GV per cell) and MDA-MB-468 cells (mean 0.7 GV per cell). The greatest number was identified in MCF10/DCIS.com cells (mean 3.0 GV per cell).

In view of the relatively large size of GV and their novelty we further characterised them in MCF-7 cells by measuring the diameter of intracellular GV in relationship to the diameter of the cytoplasm and nucleus in the same cell in 20 randomly selected cells (Figure 4C). Within this sample of 20 cells, we identified a mean of 2.55 (range 1-6) intracellular GVs per cell. The mean diameter of individual intracellular GV was 70.77% of the mean cytoplasm diameter, 116.31% of the mean nuclear diameter and 44.76% of the total cell diameter. The mean total cell diameter (including intracellular GV) was 158.09% of the mean cytoplasm diameter. This illustrates that single intracellular GV constitute a large proportion of the cell volume. Furthermore, the data also suggests that the intracellular GV volume may be greater than that of the cytoplasm volume in cells with more than one intracellular GV.

### Breast cancer cell lines produce giant extracellular vesicles

In addition to being present as intracellular vesicles, GV were noted to be present as free vesicles within the extracellular fluid. FM® 1-43FX-fluorescence imaging of MCF-7 and T47D cells identified large extracellular vesicles similar to that of the above described intracellular GV (Figure 1D). Similar large extracellular vesicles were identified using two further live cell imaging methods: DIC and FM® 4-64FX-fluorescence imaging of MCF-7 and T47D cells (Figure 2C, 2D, 2G & 2H). These extracellular vesicles were present in large numbers in the extracellular fluid and demonstrated a deformable structure when flowing in the extracellular fluid between cells (Supplementary videos 1 & 2).

### Giant vesicles are present in a patient-derived xenograft murine breast cancer model

**Figure 5:**
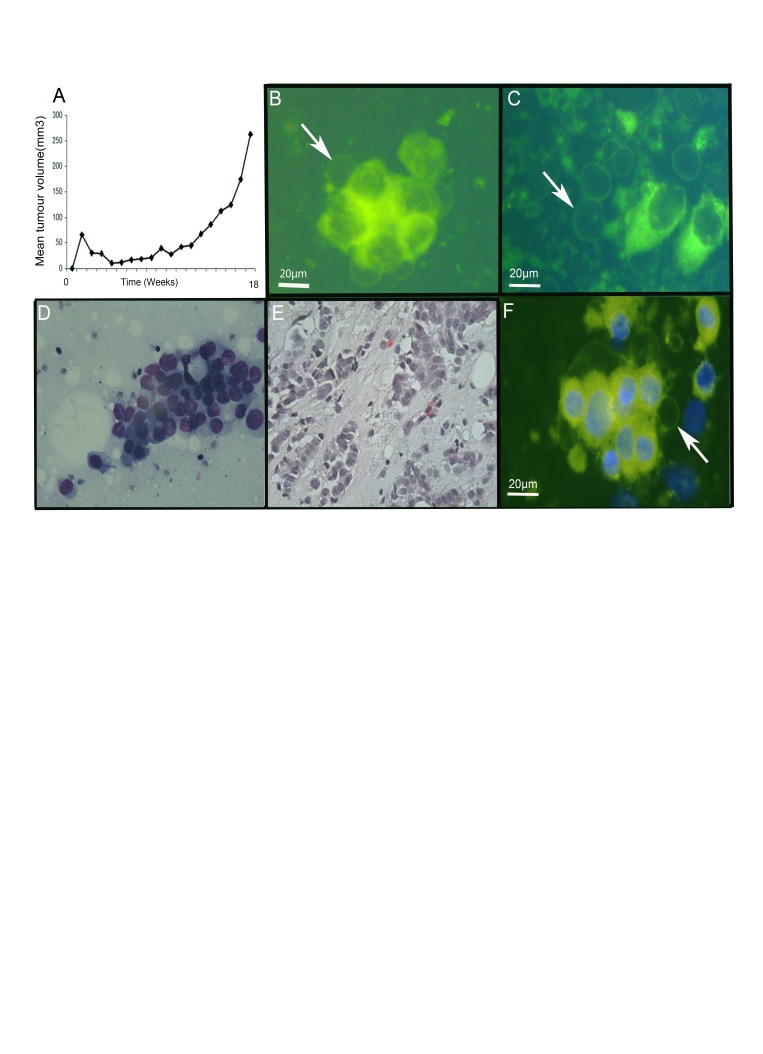
Giant vesicles are produced in xenograft and primary breast tumours Primary human tumour tissue was implanted subcutaneously into mice (5A) and growth of the xenograft (tumour volume, mm^3^) monitored over time. At 18 weeks, mice were culled and tumours removed. (5B-C) Tumour cells from the xenograft were prepared by imprint cytology, labelled with FM® 1-43FX (green) and DAPI (blue) for live cell imaging. Scale bar represents 20μm. Figure 5B illustrates intracellular giant vesicles. Figure 5C illustrates extracellular giant vesicles. (5D-F) Invasive breast carcinoma samples from patients were prepared by imprint cytology for live cell imaging with FM® 1-43FX (green) and DAPI (blue) labelling (5F) and Diff Quick staining (5D). The tumour tissue used for preparing the imprint cytology samples was then fixed in formalin and processed routinely after paraffin wax embedding prior to staining sections with H&E (5E).

We investigated a patient-derived xenograft murine breast cancer model to establish if GV were present in tumours and to exclude the possibility that they were a feature limited to cultured cells. Tumour tissue implanted subcutaneously were allowed to grow, and collected after 18 weeks of growth (Figure 5A). Similar intracellular and extracellular GV were identified by live cell imaging of imprint cytology samples labelled with FM® 1-43FX and DAPI (Figure 5B-C). This confirmed the existence of GV in this xenograft murine breast cancer model providing further evidence for the existence of GV in tumours.

### Giant vesicles are present in human invasive breast carcinoma tissue

We also investigated primary tumour samples from patients to see if GV were present in human invasive breast carcinoma tissue. We performed imprint cytology to capture breast cancer cells from two ERα-positive invasive breast carcinoma samples. Cells were immediately labelled with FM® 1-43FX and DAPI to allow live cell imaging. This identified intracellular and extracellular GV in both samples (Figure 5F). Intracellular GV were present at the periphery of cells. Free extracellular GV showed no DAPI fluorescence confirming that they did not contain a nucleus and were therefore not cells.

To confirm appropriate tissue sampling, imprint cytology prepared from the same samples were stained with Diff Quick which confirmed the presence of adenocarcinoma cells (Figure 5D). Additionally, subsequent histological examination of the tissue samples used for imprint cytology confirmed the presence of invasive carcinoma (Figure 5E).

### E2 induces giant vesicle formation in E2-responsive ERα-positive breast cancer cells

**Figure 6:**
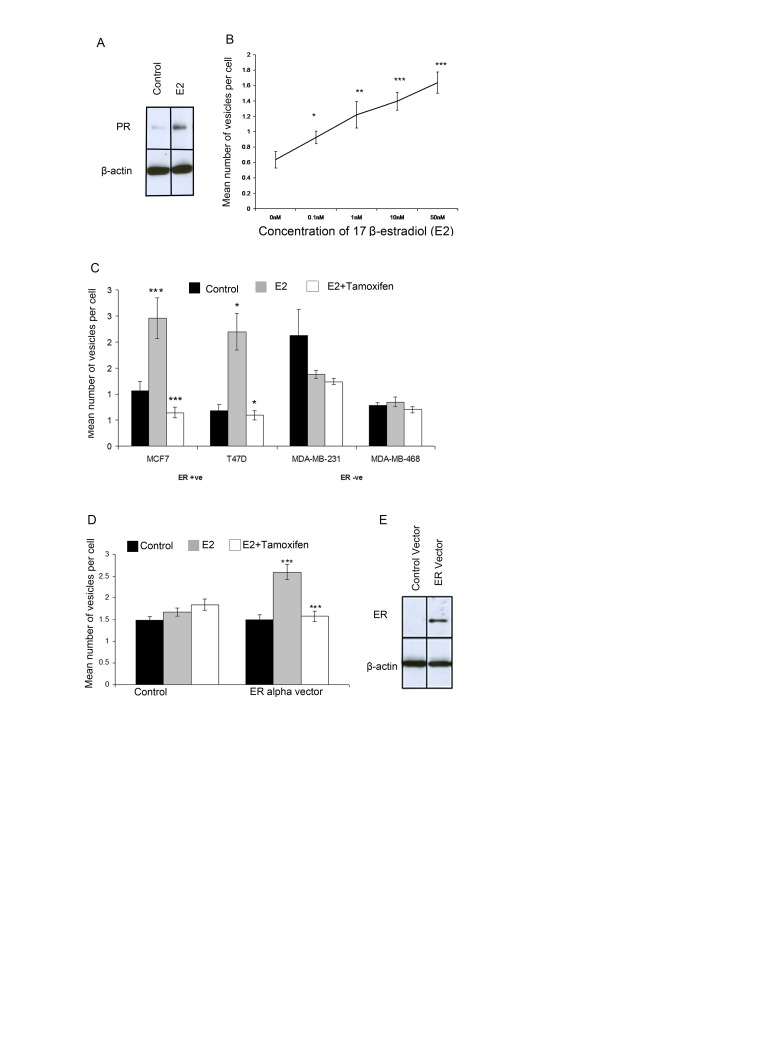
Giant vesicle production is regulated by 17β-estradiol (E2) via ERα MCF-7 cells were treated with 17β-estradiol (E2). Western blot analysis for PR expression showed adequate steroid-withdrawal and activation of E2 signalling following E2 treatment (1nM E2) Beta actin was used as a loading control (6A). The number of giant vesicles per cell was calculated and means plotted with +- SE bars. A t-test for each concentration compared to control was performed (6B). MCF-7, T47D, MDA-MB-231 and MDA-MB-468 cells were treated with 1nM 17-estradiol +/− 1nM tamoxifen and the number of giant vesicles counted per cell. Means plotted with +- SE bars (6C). MDA-MB-231 cells were transiently transfected with ER-alpha or control vector prior to treatment with 1nM 17β-estradiol +/− 1nM tamoxifen and number of giant vesicles per cell counted. Means plotted with +- SE bars. t-test to compare control with E2-treated, and E2-treated compared with E2 + tamoxifen-treated performed (, *** p<0.0001 ** p<0.001, * p<0.05) (6D). western blot analysis was used to confirm ERα expression, using beta-actin as a loading control (6E).

To investigate the role of E2 in GV formation, MCF-7 cells were withdrawn from steroids over five days. On the fifth day, MCF-7 cells were incubated with 0nM, 0.1nM, 1nM, 10nM and 50nM E2 for 72 hours before imaging using FM® 1-43FX and DAPI. To confirm that experiments involved adequate steroid withdrawal and E2-stimulation, Western transfer analysis for the E2-regulated protein progesterone receptor (PR) was performed on protein extracted from MCF-7 cells. Under steroid-withdrawn conditions PR protein expression was markedly reduced. Whereas, E2-treatment for 72hrs led to induction of PR protein expression, thus validating the experimental conditions (Figure 6A). GV formation increased with increasing E2 concentrations in a dose-responsive manner (Figure 6B). The results indicate a highly significant difference (p<0.05) between the number of GV produced at each concentration of E2 compared with the control.

To further investigate this, two E2-responsive ERα-positive breast cancer cell lines (MCF-7 and T47D) and two E2-independent ERα-negative breast cancer cell lines (MDA-MB-231 and MDA-MB-468) were withdrawn from steroids for five days followed by culture for 72 hours using steroid-depleted media (control), 1nM E2 or 1nM E2 with tamoxifen. Cells were then imaged with FM® 1-43FX and DAPI. E2 treatment led to a large increase in the number of GV per cell in both ERα-positive breast cancer cell lines compared with control samples (p<0.05) (Figure 6C). The number of GV per cell was significantly decreased following addition of tamoxifen compared with E2 treatment alone in both ERα-positive breast cancer cell lines (p<0.05) (Figure 6C). However, in ERα-negative cell lines, there was no significant difference in the number of GV produced per cell after E2-treatment compared with control samples (p>0.1) or between cells treated with tamoxifen combined with E2 compared with control samples (p>0.1) (Figure 6C).

### E2 mediates giant vesicle formation via ERα in breast cancer cells

Finally, to determine whether the effects of E2 on GV formation were mediated via ERα, the ERα negative MDA-MB-231 cell line was transfected with a DNA ERα vector prior to steroid withdrawal over three days. ERα transfection was confirmed to be successful by identification of ERα protein in transfected cells by western transfer analysis compared with a lack of ERα protein expression in cells transfected with the control vector (Figure 6E).

Transfected cells were then cultured for 72 hours under control or treatment conditions. This demonstrated that there was a significant increase in GV per cell in the cells transfected with ERα vector that were treated with E2 (p<0.05) (Figure 6D). Treatment with E2 together with tamoxifen in these ERα transfected cells led to a significant decrease in the number of GV per cell (p<0.05) (Figure 6D). There was no significant difference in the number of GV per cell between the three conditions (control and treatments) in cells transfected with the control vector (Figure 6D). These results indicate that the effects of E2 and tamoxifen that lead to increased or decreased formation of GV respectively in breast cancer cells is mediated via ERα.

## DISCUSSION

In the present study we have provided strong evidence for the existence of large intracellular and extracellular vesicles (GV) in breast cancer. The diameter of GV ranged from 3μm to 42μm. The principal imaging modality used in this study (FM® 1-43FX fluorescence) was validated using two additional independent live cell imaging techniques, confirming the existence of GV in breast cancer cells and validating FM® 1-43FX for further use as a tool to detect GV. GV were also identified in invasive breast cancer tissue from both a xenograft murine breast cancer model and from excision specimens. This suggests that GV exist in invasive breast carcinoma tumours and that they are not simply an *in vitro* phenomenon.

The diameter of intracellular GV can be similar to that of the cytoplasm component of the cell. Furthermore, GV contribute to a large proportion of the cell volume, especially in cells with multiple GV. In this regard, GV represent an important aspect of cancer cell cytomorphology. The extracellular GV described in the present study are the largest described in the literature to the authors' knowledge at the time of writing and are many times larger than the submicron diameter microvesicles that have now been extensively studied

The identification of GV in breast cancer is supported by the work of Di Vizio *et al* who recently described extracellular vesicles 1-10μm in diameter produced by prostate cancer cells [36]. These vesicles were shown to contain bioactive matrix metalloproteinases (MMP2 and MMP9), enzymes that have critical importance in tumour invasion, in addition to RNA, ADP-ribosylation factor 6 and caveolin-1. Additional evidence for the existence of extracellular vesicles larger than submicron diameter microvesicles is also provided by the recent identification of extracellular vesicles up to 8μm in diameter produced by astrocytes [37].

We have also shown that GV formation occurs in an E2-responsive manner that is repressed by tamoxifen in ERα-positive breast cancer cells. We have also provided evidence that this action of E2 is mediated via ERα. However, GV are also produced by ERα-negative breast cancer cells but as an E2-independent phenomenon. This suggests that GV are a general feature of both E2-dependent and E2-independent breast cancer with the vesicle trafficking proteins involved in their formation being under E2-regulation in the former. As the action of E2 is instrumental in E2-dependent breast cancer, the E2-regulation of GV formation suggests that GV may have an important role in breast tumourigenesis. These findings are supported by the fact that E2 also regulates vesicle trafficking gene expression in breast cancer cells, in addition to the ultrastructural study of Vic *et al* described above [6,7]. The results of the present study taken together with the ultrastructural findings of Vic *et al* suggest that E2 regulates the formation and release of vesicles of a range of sizes including of submicron diameter, in addition to the GV that we have identified. In the same era as the Vic *et al* study, Tulusan *et al* investigated the ultrastructure of invasive breast cancer tissue samples and found a significant association between intracytoplasmic vacuoles and ERα-positive status [38]. As vacuoles are a form of intracellular vesicles, this provides some additional evidence for a link between E2 and vesicle trafficking in breast cancer. Further support for the hormonal link with vesicles is provided by studies in the DBA/2Cr mouse which have shown that large vacuole formation in renal tubule cells is under the regulation of sex steroid hormones [39]. It is also interesting, that there is a body of evidence supporting E2 as a regulator of exocytosis by non-genomic actions [40].

The existing evidence for the importance of vesicle trafficking and extracellular microvesicles in cancer tends to suggest that GV themselves are likely to have an important role in breast cancer. In the present study, variable numbers of GV per cell were identified in different cell lines, including the non-neoplastic breast epithelial cell line MCF-10A. Although MCF-10A is an immortalised cell line, these results do provide some evidence for the existence of GV in non-neoplastic breast epithelial cells. Therefore, GV formation could potentially be process that occurs in normal cells under physiological conditions, as well as in tumourigenesis.

Extracellular GV may represent a previously unknown population of large extracellular vesicles with similar functions to the well characterised principally submicron diameter microvesicles. Therefore, extracellular vesicles are likely to exist as a spectrum of diameters from the smallest diameter of previously described extracellular vesicles including exosomes, up to the largest GV diameter (i.e. from around 30nm up to at least 40μm). As GV are released into the extracellular fluid, this suggests that their formation is related to exocytosis or a similar vesicle trafficking process. GV release may represent an important mechanism of cellular secretion. As many proteins (such as MMPs, CXCL12 and VEGF) that are important in breast cancer are secreted, this could be highly significant.

In summary, in the light of the E2-regulated transcriptome including a large number of vesicle trafficking genes, we have investigated the effect of E2 on vesicles in breast cancer cells using live cell imaging. Over three decades after the publication of the elementary ultrastructural study of Vic *et al*, we report a re-investigation of this field which has identified large vesicles of unprecedented size: giant vesicles (GV). The results of future studies investigating the functions of GV in breast cancer are awaited.

## METHODS

### Cell lines

Four ERα-positive breast cancer cell lines (BT-474, MCF-7, MCF7-HER218, T47D), two ERα-negative breast cancer cell lines (MDA-MB-231, MDA-MB-468), two breast ductal carcinoma in situ (DCIS) (MCF10DCIS.com and SUM225) cell lines and one non-neoplastic breast epithelial (MCF-10A) cell line were used in this study. ERα-positive cell lines were cultured in DMEM. ERα-negative cell lines were cultured in RPMI. Both media were supplemented with 10% foetal bovine serum (FBS). MCF10A cells were cultured in DMEM F12 with 5% horse serum, epidermal growth factor (EGF) (20ng/ml), hydrocortisone (100ng/ml), insulin (1µg/ml) and cholera toxin (100ng/ml). SUM225 cells were cultured in Hams F12, 5% FBS, insulin (5μg/ml), HEPES (50μg/ml) and hydrocortisone (1µg/ml). MCF10DCIS.com were cultured in DMEM:F12, advanced with 5% horse serum. All cell lines were supplemented with 50μg/ml L-glutamine and 50µg/ml penicillin/streptomycin (P/S).

### Invasive breast carcinoma samples

Breast tumour samples were collected from patients with primary ERα-positive invasive breast carcinoma. Fresh tumour samples from excision specimens were obtained immediately after surgery and transported to the research laboratory in DMEM prior to preparation for live cell imaging and Diff Quick staining using imprint cytology. After preparing imprint cytology slides, tissue samples were fixed in formalin (4% formaldehyde) and processed using standard histology techniques to produce formalin-fixed, paraffin wax-embedded tissue sections that were stained with haematoxylin and eosin.

### Xenograft-derived breast cancer cells

Fresh invasive ductal breast carcinoma tumour fragments, confirmed to be ER+/PR+ and measuring between 2 × 2mm and 3 × 3mm, were grafted immediately after removal, into the interscapular fat pad of 8–12-week-old female Swiss nude mice under general anaesthesia and veterinary control. Mice were maintained in a specific pathogen-free animal housing (Institut Curie) and received estradiol (E2, Sigma-Aldrich, Saint-Quentin Fallavier, France) diluted in drinking water (8µg/ml) until engraftment was confirmed. After a latency period tumours appeared at the engraftment site. Once the tumour had reached a critical size of about 2500mm^3^, mice were sacrificed, prior to removal of the tumour. Tumour was dissected into 2–3mm diameter fragments and re-engrafted in nude mice fed with E2-containing water for sequential passages. A xenograft was deemed as established when it has been passed through three consecutive *in vivo* passages [41]. Imprint cytology was used to harvest tumour cells for live cell imaging.

### Ethics Statement

Any experimental research reported in the manuscript has been performed in compliance with the Helsinki Declaration and according to national and international guidelines and has been approved by the authors' institutional review board. All samples were collected with informed written consent. Patient derived breast cancer cells from tumour samples were obtained from the Manchester Cancer Research Centre (MCRC) Biobank, UK (project ID: 09_GOLA_02). The role of the MCRC Biobank is to distribute samples and therefore, cannot endorse studies performed or the interpretation of results. Established primary human xenograft models were obtained from the Institut Curie with ethical approval from an internal review board and informed written consent of patients [41] and experiments performed in the UK with a home office approved project licence (PPL40/3645).

### Imprint cytology of tumour samples

To obtain cells for imaging from primary and xenograft tumour samples, the tumour was washed in room temperature Hank's balanced salt solution (HBSS) (H9394, Sigma-Aldrich). A sterile scalpel blade was used to slice thin sections through the tumour. The cut surfaces of the tumour were then pressed gently onto a microscope slide for a few seconds. Cells were then labelled with FM® 1-43FX and DAPI for live cell imaging as described below. Additional slides from each primary tumour sample prepared at the same time were stained using a modified Giemsa stain according to manufacturer's instructions (Diff Quik Stain Kit, Polysciences Ltd).

### Fluorescent labelling for live cell imaging

Tumour cells were labelled directly immediately after imprint cytology slides were prepared. Cell lines to be imaged were plated in a monolayer of 40,000 cells per 1.7cm^2^ chamber on a four-well microscope slide. Cells were grown in appropriate media specific to their cell line. Cells were washed with HBSS (H9394, Sigma-Aldrich) prior to addition of 40µL of ice-cold 5µg/µL FM® 1-43FX (F35355, Invitrogen) or 5µg/µL FM® 4-64FX (T13320, Invitrogen) in HBSS for one minute. Cells were mounted with 40µL of DAPI mountant (H1200, Vector Laboratories) and incubated at room temperature for ten minutes before imaging.

### Fluorescence microscopy

Fluorescent live cell imaging and photography was performed using an Olympus CKX41 inverted light microscope with an X-cite 120 fluorescence camera and illumination system. Images were captured for 13 fields of views and the number of cells per field of view (x400 magnification) was counted, as determined by nuclear DAPI staining. Using FM® 1-43FX fluorescence, the number of giant vesicles (GV) (greater than 1µm in diameter) was counted in the same fields. Imaging was also performed using an Olympus IX81 with spinning disk confocal microscope and images taken using an Andor iXon DU888 camera.

### Differential interference contrast microscopy live cell imaging

MCF-7 and T47D cells were grown in media containing 10% FBS for 48 hours in chamber slides prior to imaging. The medium was removed and replaced with room temperature phosphate buffered saline (PBS) solution 15 minutes before imaging. Differential interference contrast (DIC) microscopy was performed using a Zeiss Axiovert 200M motorized inverted time-lapse microscope with a 100X oil immersion lens. Images were collated using Metamorph and Image J imaging software.

### Time lapse microscopy live cell imaging

MCF-7 cells were plated for time-lapse photography in a 24-well glass bottomed plate and washed twice with PBS before 50µL complete DMEM media was added. DIC microscopy was performed using a Zeiss Axiovert 200M motorized inverted time-lapse microscope with a 100X oil immersion lens. The microscope was contained within an environment chamber, allowing cells to be incubated at 37°C and 5% CO_2_. The time lapse images were taken at ten second intervals using a Photometrics Cascade 512B camera. Images were collated using Metamorph software.

### Apoptosis and necrosis assay

Annexin V-FITC, an apoptotic marker, and propidium iodide (PI), a necrosis marker, were used to compare the levels of apoptosis and necrosis respectively in breast cancer cell lines in relation to the number of cells producing GV. Cells were re-suspended in 500µl 1X Annexin V binding buffer with 5µl of Annexin V-FITC and 5µl PI, incubated for 15 minutes at room temperature, fluorescence detected using a Becton Dickinson FACS Calibur, data processed using BD CellQuest™ Pro V6.0 and analysed using WinMDI V2.8 software.

### 17β-estradiol and tamoxifen treatments

For all experiments to assess estrogen signalling, charcoal-stripped serum was used. Dextran coated charcoal (250mg) (C6421, Sigma Aldrich) was added to FBS (25ml) before heating at 55°C for 30 minutes in a water bath. The mixture was centrifuged (2000g for 15 minutes) to separate the charcoal from the solution before sterilising using a 0.22µm filter and storing at -20°C. Cells were withdrawn from steroids present in serum over five days decreasing from 10% serum to 1% serum in phenol red-free media, prior to treatment and analysis. MCF-7 cells were treated with increasing concentrations of 17β-estradiol (E2) (E2758, Sigma-Aldrich) (0.1nM, 1nM, 10nM and 100nM) for 72 hours. MCF-7, T47D, MDA-MB-231 and MDA-MB-468 cells were treated with 1nM E2 with and without 1nM tamoxifen for 72 hours (T5648, Sigma-Aldrich). FM® 1-43FX and DAPI fluorescent live cell imaging was then performed.

### Transient transfection with Estrogen Receptor Alpha

4 × 10^5^ cells were seeded in a 28.3 cm^2^ culture dish with RPMI for 24 hours. The media was subsequently removed and P/S-free SFM (serum-free media) added along with 1ml of solution containing either 3µg pVP16-ER vector (Addgene, #11351) or pVP16 only (control) (donation from Keith Brennan) with Opti-MEM® (Gibco, #11058021) and Lipofectamine® 2000 (Invitrogen Life Technologies, #11668-019). Six hours after transfection, SFM was replaced with SM (serum media) and cells were allowed to grow for 24 hours before subsequent analysis. All transfections used Lipofectamine® according to the manufacturer's instructions.

### Western Blotting

Protein was quantified using BCA assay (23225, Thermo Scientific) and loaded onto an SDS–polyacrylamide gel prior to transfer to a Hybond-C Extra nitrocellulose membrane (Amersham, GE Healthcare, Life Sciences, #RPN303E). Primary antibodies used were SP1-ERα (Thermo Fisher Scientific Inc., #RM-9101-SO), Progesterone Receptor (PR) (Dako, # M3569) and actin (Santa Cruz Biotechnology, #sc-1616).

### Statistical analyses

A two-tailed student's t-test was used to compare the number of vesicles produced per cell by cell lines, treatment groups or exposures. A p-value of p<0.05 was considered significant. Significance of Student's t-test is displayed in figures as *** p<0.0001, ** p<0.001 and * p<0.05.
